# Promoting mental wellbeing among youth Australian Rules footballers through a model of continuous improvement

**DOI:** 10.3389/fspor.2024.1189933

**Published:** 2024-08-09

**Authors:** Nicky Couston, Erin Hoare, Kate Hall

**Affiliations:** ^1^ Australian Football League, Melbourne, VIC, Australia; ^2^ School of Medicine, Barwon Health, IMPACT—The Institute for Mental and Physical Health and Clinical Translation, Deakin University, Geelong, VIC, Australia; ^3^ School of Psychology, Deakin University, Burwood, VIC, Australia

**Keywords:** mental well-being, young high-performance sport, positive psychology, elite sport, adolescent

## Abstract

The developmental period of adolescence is a known transitional life phase with unique risk and protective factors that can affect mental wellbeing outcomes. This, in combination with the pressures and demand of elite sport, make young elite athletes an important population in which positive mental wellbeing can be explored. This study aimed to examine the state of wellbeing, informed by positive psychology and the Positive emotions, Engagement, Relationships, Meaning, Accomplishment (PERMA) model of wellbeing, in a cohort of young athletes aged 16–19 years in the Australian setting as part of a model of continuous improvement. The objectives of these aims were to inform the development of a wellbeing curriculum for implementation in the Australian Rules Football talent pathway and to assess whether wellbeing presents differently in young athletes relative to the general school-attending population of that same age. Participants were 608 young Australian footballers participating in the developmental talent pathway during 2020 and 2021. There were 299 young male footballers, and 309 young women footballers who completed data collection. Wellbeing was assessed using the Engagement, Perseverance, Optimism, Connectedness, Happiness Measure of Adolescent Wellbeing (EPOCH) which corresponds to the PERMA model of wellbeing, and total mean scores were reported. Engagement and connectedness were similar for the young men and women athletes. Young men reported higher perseverance, optimism, happiness, and overall wellbeing relative to young women. Wellbeing among young athletes appears similar to the general population, however perseverance may be higher among young athletes. These findings form an important component of the continuous improvement model adopted in the football program in that the results informed the development of a tailored wellbeing curriculum program that is reflective of the wellbeing needs of the young athletes.

## 1 Introduction

### 1.1 Mental health and wellbeing in elite sport

The mental health and wellbeing of elite athletes is widely recognized as an important area for a range of individual, health, high performance, and other outcomes (1, 2). Highlighting this was the 2019 International Olympic Committee Consensus Statement for Mental Health in Elite Athletes, which identified that whilst mental health problems are common among athlete populations, there are likely additional sport specific factors that can contribute to the presentation and trajectory of mental ill health (3). The recognition for early intervention and mental health promotion to offset mental ill health risks in athlete populations is now widely accepted as an important area of investment (4, 5). However, there has been a lack of implementation and uptake of evidence informed frameworks in elite sport (1). Further work is needed to understand the context specific barriers to mental health promotion across sports internationally.

### 1.2 Mental ill health vs. mental wellbeing

It is broadly accepted that optimal mental wellbeing includes both the absence of mental ill health (such as common mental disorders including depression and anxiety), as well as experiencing positive wellbeing such as optimism, positive relationships with others, and purpose (6). This assumption fits within the dual continuum model of mental health and wellbeing that holds that mental illness and positive mental wellbeing are separate, but related, constructs (7). There is a body of evidence to support the investment in preventing mental ill health whilst also providing opportunities to promote mental wellbeing (8). The field of positive psychology focuses on the promotion of mental wellbeing as a standalone target, and research increasingly indicates pursuit such as engagement, purpose, optimism, and positive relationships and other indicators can lead to a range of healthy developmental outcomes (9). The primary target of positive psychology focused interventions has been within school settings, thus leading to the emergence of the sister field of positive education (10). Indeed, there is evidence to suggest fostering mental wellbeing in school-based settings may equip young people with the psychological, social, and other skills to support their development in the future (11). Whilst schools undoubtedly offer prime implementation opportunities to target large groups of young people it is noted positive psychology informed initiatives for young people outside the education setting are relatively few and may form an important area of future research.

### 1.3 Epidemiological research in elite sport

There is recognition of the importance of mental health promotion in elite athlete populations, however there appears to be a paucity of epidemiological research in this unique population (4). A narrative systematic review published in 2016 synthesizing evidence for mental health among elite athletes found 60 studies reporting on incidence and trajectories of mental ill health and wellbeing (4). Findings identified a range of vulnerabilities among elite athletes relating to mental ill health including anxiety, body image concerns, substance use, anger, aggression, and stress and coping strategies. It is noteworthy that whilst inclusion criteria included wellbeing-focused research, very few studies appeared to be dedicated to this domain. It is proposed this lack of epidemiological understanding of this unique group may adversely affect the implementation of appropriate, evidence informed, mental health frameworks, within which athletes can thrive.

### 1.4 Young athletes

Of the limited existing research to date, there is even fewer focused studies on the developmental needs in relation to mental wellbeing for young elite athletes. The young athlete experiences both the elite athlete specific demands, alongside the developmental trajectories that characterizes adolescence to adulthood (12). The myriad of challenges faced during adolescence are well understood and include biological maturation, increasing independence, and exposure to risk behaviors (13). Among young athletes the evidence to date suggests similar risks to non-athletes in terms of mental illness including anxiety, depression, substance and eating disorders (14). However, youth athletes also experience unique risk factors such as balancing education alongside training programs, social identity concerns, and overtraining. Further reviews have identified factors such as inadequate sleep, reduced family time, and social isolation as additional considerations for young athletes (15, 16). It is noteworthy that of the limited research among young elite athletes, the focus has predominately been on specific risk factors for this population in terms of developing mental ill health. There is very little research in terms of understanding key factors known to contribute to positive mental wellbeing. Further, it has been acknowledged that whilst research is directed towards understanding successful transition out of elite sport (i.e., retirement) there is little research exploring transition into elite sport as a young adult (17). We note recent calls for preparing young athletes for the transition into elite sport in terms of addressing the wide ranging, complex factors that exist at the individual, relational, sport-specific, and sociocultural levels (17).

### 1.5 Australian football and wellbeing

Belonging to sporting communities has been proposed as a powerful means of promoting wellbeing (18). Improved mental health outcomes such as lower depressive and anxiety symptoms and improved life satisfaction have been observed among youth who participate in sports, alongside a host of other positive developmental outcomes (19, 20). Importantly, there is evidence to suggest this relationship has been identified to be mediated by peer belonging (20). In Australia, Australian-Rules Football (henceforth AFL) being one of the highest ranked sports in terms of national participation and spectatorship, may play a critical role in facilitating wellbeing by building connection, enhancing support networks, providing opportunity to education on leadership and contributing to team goals, and other protective factors. Australian Rules football clubs benefit from the opportunities provided by community clubs generally, and also the unique factors that characterise Australian football which has been acknowledged as an important setting for social change (21, 22). It also benefits from investment in mental health and wellbeing with the national governing body of the sport committing to co-ordinated, systemic action in terms of promoting mental health and wellbeing through the AFL Industry Mental Health and Wellbeing Strategy (23). The above factors combined make Australian football a potentially enriching environment for young people, and thus of great interest in terms of scholarly research in mental wellbeing.

### 1.6 Model of continuous improvement

Improving sustainability, resource utilization and appropriateness of interventions is best supported through models of continuous improvement (24). Continuous improvement is the process through which data are collected on interventions or initiatives, results are assessed, and changes are made as a result of such data analysis and subsequently scaled up to populations (25). Failure to incorporate continuous improvement can increase the risk of inconsistency between program objectives and participant outcomes. Including continuous improvement supports a systematic and coordinated approach to intervention improvements, through which real-time observations and data surveillance are used to change and improve efforts (26). Mental health and wellbeing promotion has been described as optimally achieved through models of continuous improvement, and is particularly important for community-led initiatives which may face lower adherence to evidence-based practice in comparison to traditional scientific research methods (27, 28).

In the current study, continuous improvement is adopted in the wellbeing programs within the AFL Industry to inform program design, and to understand how to best meet the ever-changing needs of athletes throughout development programs. As an example (and the data source for this current study), the collection of wellbeing data occurs annually in the AFL talent programs, for the purpose of ensuring the wellbeing initiatives are appropriately designed and data informed to best support the unique needs of the youth football cohorts. This model is a critical component of the program development, given the extensive training schedule to which the young athletes participate, and the need for wellbeing initiatives to be pragmatic, appropriate, and effective within the time allocated to this component of their comprehensive football program.

### 1.7 Context

The AFL Talent program has previously been described elsewhere (23) however briefly, the Talent program comprises the nationally identified talent athletes for training and competition with the prospect of drafting into the men's professional program, and the women's program which is currently semi-professional. There are approximately 1,500 athletes identified annually who are invited to participate in the talent programs which includes a dedicated wellbeing program informed by positive psychology (23). We aimed to assess wellbeing through the positive psychology informed measure EPOCH, which corresponds to the PERMA model of wellbeing (29). This measure, further described below, assesses wellbeing through domains engagement, perseverance, optimism, connectedness, and happiness. This model of wellbeing is designed to assess the positive characteristics during adolescence that can lead to domains of wellbeing in adulthood (29).

As described, this study was used in a continuous improvement framework to inform wellbeing curriculum development and delivery, as part of a broader wellbeing model and program being implemented in the AFL Talent Pathway environment. Continuous improvement occurs through various mechanisms in the AFL Talent Pathway, including evidence reviews to ensure practices align to new and emerging science, on-going process related feedback from those involved in the AFL Talent Pathway programs, and through a live underpinning logic model that is revised as required to meet the evolving needs of the athlete community. Specifically, the process was undertaken to understand the athletes’ wellbeing profiles as represented in EPOCH, to ensure their wellbeing needs are met in a targeted and informed way through the wellbeing program. Thus, data collection is an annual initiative and occurs to further understand which program components would be of most benefit to the wellbeing of athletes in these pathways programs. Whilst the EPOCH measure has been used among large cohorts of adolescents, this has almost exclusively been in the school and education contexts (30). To our knowledge there is yet to be a large-scale assessment of wellbeing among youth elite athletes, informed through positive psychology, and specifically for the use in a model of continuous improvement. It is proposed that understanding these domains will help to understand the specific characteristics of young athletes in terms of wellbeing and support the refinement of wellbeing focused initiatives that seek to promote outcomes among this unique population group.

### 1.8 Study aims

The aim of this study was to report on the mental wellbeing, informed by positive psychology, among a group of Australian young people currently competing in the Australian Football League (AFL) Talent program for the purpose of continuous improvement. We aimed to conduct preliminary exploratory assessment as to whether youth elite athletes differ to adolescents in an education setting, thus require bespoke programs and initiatives in relation to wellbeing that are fit for purpose.

## 2 Methods

### 2.1 Study design

The Australian Football League (AFL) context has been described previously (23). Briefly, however, the AFL is the national Australian professional football competition which includes the pre-eminent national men's and the women's leagues. The AFL's National Talent Pathway is the program for talent-identified adolescents (16–19 years) who participate in the high-performance leagues and academies throughout the country. There are approximately 1,500 young athletes that are representative of their regions who participate annually in the National Talent Pathway programs in preparation for potential drafting to the men's (AFL) and women's (AFLW) professional leagues.

Whilst a large focus of these programs is physical high-performance and competition, there is also a series of programs and dedicated resources for broader athlete development (23). This includes a specific wellbeing program designed to build the characteristics that can support current and future healthy development which commences at the enrolment in the high-performance program and concludes at the end of the football training program 10–12 months later. Athletes commence the wellbeing program upon their commencement of the physical athletic training schedule annually. The specific components of the wellbeing program are described previously (23). The evaluation of the wellbeing program is forthcoming. During the initial intake into these programs, during which the young people complete a range of administrative and other data collection, the young athletes are invited to participate in anonymous wellbeing data collection. This data collection is used to inform the development of priority areas within the wellbeing program and forms a component of standard practice of evaluation and continuous improvement for the AFL.

The use of this data for the purpose of this current study has previously received ethics approval from Deakin University Human Research Ethics Committee (HEAG-H 144_2021).

### 2.2 Measures

The EPOCH Measure of Adolescent Wellbeing is based on the PERMA model of wellbeing which identifies positive emotion, engagement, relationships, meaning and accomplishment (29). It is proposed that these domains contribute to overall wellbeing and are subsequently worthy pursuits in terms of optimal mental wellbeing. The EPOCH measure is adjusted to recognize the unique developmental needs of adolescents. EPOCH corresponds to engagement which refers to being involved in activities and the environment. Perseverance refers to the pursuit of a goal despite setbacks or challenges. Optimism refers to holding hope in regards to the future. Connectedness refers to being supported and valued by others, not merely the presence of others but the closeness of relationships. Happiness refers to both the emotion of being happy, but also the experience of being content with life.

The EPOCH is a 20-item measure which has demonstrated good reliability, convergent and divergent validity, and predictive validity (29). Each item is scored on a Likert scale ranging from almost never (=1) to almost always (=5), with higher scores demonstrating higher levels of wellbeing in the domain. Subscale scores are calculated based on average scores from the four corresponding items. A total EPOCH score is also calculated reflecting general overall wellbeing.

EPOCH measures were delivered in the initial intake of athletes in their respective talent development teams at the commencement of the pre-season. Athletes competed the measure online, and all data were anonymous. Athletes were provided introduction to the measure through describing the purpose of the data were to understand wellbeing and to help inform the subsequent tailoring of programs to meet the young people's needs.

### 2.3 Statistical analysis

All statistical analyses were undertaken using Stata. Little's MCAR test (31) was conducted using Stata command (mcartest) to determine the impact of missing data on the analysis. Linear regression models, whereby the domain of wellbeing was treated as a continuous score as the dependent variable, were used to assess whether young athletes gender predicted outcomes scores across each domain of wellbeing. Analyses were adjusted for the clustering effect of team in which the athletes competed. Aggregate (mean and standard deviations) were sought from the original EPOCH study to further understand the current findings in the context of what is known about the general adolescent population (29). We have accepted *p* < 0.05 as statistical significance, however in the results tables we report exact *p*-values and 95%CI to allow for interpretation of the data (specifically correction for multiple comparisons was not completed due to the exploratory nature of this study).

## 3 Results

A total of 608 young athletes participated in data collection, which included 299 boys and 309 girls (Table 1). Approximately 60% were from non-metropolitan areas, and 40% were based in metropolitan geographic regions (Table 1). Within the boys, the highest mean score in mental well-being appeared to be connectedness, relative to other domains of mental wellbeing (Table 2). This was similarly found within the girls' cohort, in which the highest mean was also in the connectedness domain (Table 2).

**Table 1:** Participants characteristics.

	*N*	%
Gender		
Boys	299	49.2
Girls	309	50.8
Location		
Metropolitan	248	40.9
Non-metropolitan	358	59.1

May not add to total due to small number of missing data.

**Table 2:** Mean and SD of wellbeing domains, as measured by EPOCH by boys and girls.

	Boys (*n* = 299) M(SD)	Girls (*n* = 309) M(SD)	Total (*n* = 608) M(SD)
Engagement	3.56 (0.75)	3.50 (0.79)	3.53 (0.77)
Perseverance	4.19 (0.58)	4.05 (0.67)	4.12 (0.63)
Optimism	3.66 (0.73)	3.51 (0.76)	3.59 (0.75)
Connectedness	4.40 (0.60)	4.39 (0.64)	4.39 (0.62)
Happiness	4.04 (0.70)	3.91 (0.81)	3.97 (0.76)
Overall	19.85 (2.47)	19.35 (2.77)	19.60 (2.63)

Upon comparing boys and girls, perseverance, optimism, happiness, and overall mental wellbeing were observed to be higher among boys than girls (Table 3). Whereas engagement and connectedness were similar. Optimism and perseverance appeared to have the greatest difference between boys and girls. Whilst no statistical comparisons were made, school attending adolescent data reflective of the wider Australian population (Supplementary Table 1) suggested young elite athletes tended to report higher levels of mental wellbeing relative to the general population of the same age (29).

**Table 3:** Linear regression coefficients for each wellbeing domain (DV), with predictor gender where boys = 0 and girls = 1, adjusted for clustering effects of team.

	*b*	95%CI	*p*
Engagement	−0.07	−0.20, 0.06	0.284
Perseverance	−0.15	−0.25, −0.04	0.012
Optimism	−0.15	−0.26, −0.04	0.011
Connectedness	−0.02	−0.14, 0.10	0.728
Happiness	−0.14	−0.27, −0.01	0.042
Overall	−0.52	−0.97, −0.07	0.028

## 4 Discussion

This study assessed mental wellbeing among a large cohort of Australian elite youth athletes, participating in the AFL National Talent program as a component of continuous improvement within a wider wellbeing program and curriculum. This study is consistent with recent calls to action that articulate that youth athletes compete in highly specialized contexts and represent a critically under researched population (2). This is particularly so given the COVID-19 context which has been recognized as a chronic stressor to which has had significant consequences for the wider population to which high performance athletes were not excluded (32). The athletes in the present study reported high levels of connectedness, and boys tended to report higher levels of mental wellbeing across most domains relative to girls. It may be that young athletes reported higher levels of mental wellbeing relative to the same age general population, however no statistical comparisons were made to support this hypothesis. The current findings support bespoke wellbeing programs for young athletes that are separate and distinct from education settings and further justify the need for specific consideration of the wellbeing needs of young men and women participating in talent development pathways. Sport specific risks to mental wellbeing could be bolstered through the PERMA approach, which has had widespread uptake in education sector but is yet to be evaluated in the sport setting.

The relationship between peer belonging and connectedness and team sports participation has been previously reported (20, 33). This has specifically been the case for social connectedness in the context of adolescents, who typically place increased value on the views and perceptions of their peers (12). It has been suggested that the increased value of peer-based relationships can be harnessed through sport which typically requires group cohesion to achieve goals (34, 35). It is also likely that the relationship between social connectedness and sports participation is bi-directional. For example, whilst sport provides opportunity for social interaction and increased connectedness, it is also possible that those young athletes who successfully engage in higher levels of connectedness may be more likely to excel in sport which requires investment in teammates and the wider sporting team (33).

Young male athletes tended to report higher levels of wellbeing relative to young female athletes, which is consistent with the wider evidence base suggesting gender differences in mental wellbeing (36, 37). We note the exploratory nature of these analyses thus further research is needed to confirm this relationship. However, there are a range of theories and rationales to explain this difference. There are known social and environmental risk factors that may differentially affect male and female young people. For example, there appears to be increased societal pressures to subscribe to normative roles with the onset of puberty which may disproportionally affect young women's risk, who typically enter puberty before young men (38). Relational theories focus on the roles of young men and women and the peer relationships that develop during adolescence, which may explain differences in mental wellbeing (39). Australian women's football just recently became elite and semi-professional, and thus the socio-cultural-historical context for young women athletes cannot be overlooked. Whilst this study is cross-sectional thus directionality cannot be inferred, it could be argued that the existence of differences in profiles among the young athletes is not surprising in the context of such circumstances.

There is an argument that to succeed in high level sport, individuals must possess skills that enable them to meet the unique requirements of competition such as perseverance and optimism (40). It is also possible that such skills are fostered through participation in elite programs, in which the athlete development occurs both on and off field such as through performance pressure, media scrutiny, and through meeting the demands of both education and sport (4). Given such arguments, it is not surprising that the wellbeing profile of athletes in this study differed to that of the same-age population in the general population. Whilst outside the scope of this study, understanding the developmental trajectories of athletes compared to non-athletes is of interest for future research. Such insights could hold important performance related implications, and for the developmental outcomes of young athletes entering, competing, and eventually transitioning out of professional competitions. Mental wellbeing promotion for youth in the general community is rapidly evolving in the education sector (41), and the current findings suggest that targeted programs may be needed for young athletes who present with different wellbeing profiles to that of the general community. We note the potential for these findings to be generalized to other sporting codes, given some of the characteristics of high-performance young people (42) which have previously be suggested to be homogenous.

### 4.1 Limitations

Whilst the current study adds to the young athlete mental wellbeing evidence to which there is a current need, there are several limitations to be considered. Firstly, the data collected in this study formed a part of continuous improvement activities, and therefore the scope and detail in the study design were limited. This limits the extent to which these data can be reliably used to draw rigorous conclusions. Whilst limited, we do acknowledge these data provide insights into a relatively underrepresented study cohort and these findings may be used as a basis from which further scientifically rigorous data are collected. In particular, the level of detail relating to participants was limited and as was the subsequent level of analysis that was achieved. As such, the data were not able to be adjusted for factors other than team with which the athlete competed. The data were also not able to be analyzed in terms of how represent the participants were in terms of ethnicity, level of education, health status, and other key factors that may have been of interest in the context of mental wellbeing. These data were collected during the COVID-19 pandemic which is acknowledged to have had significant impacts on health research data collection methods globally to which the current study is also likely to have been affected (43).

Whilst these findings are expected to contribute to youth athlete mental wellbeing generally, it must be considered that the participants were current participants in the Australian football talent program, and the extent to which the findings extend to other sports and sectors is not known. The application of positive psychology to high performance sport is supported by a growing body of evidence (44, 45), however we note the relatively limited evidence to date for the application of such approaches for young high-performance athletes (23). Thus we propose this study, and the program more widely, forms an important advancement is the area to date. A final limitation is the EPOCH measure, whilst validated in general population settings (29), is yet to be validated among athlete cohorts thus further limiting these findings.

### 4.2 Implications and future directions

There is a need to understand the needs of young athletes and particularly the mental wellbeing experiences of young people in talent development pathways, as they prepare to enter professional sport. It is proposed that understanding mental wellbeing during these developmental years may allow the offsetting of risk factors that may increase the likelihood of mental ill health and support the overall development of the individual athlete. Adopting population-based assumptions, such as wellbeing of young people in education settings reflect that of those who participate in elite-level youth sport, may hinder efforts in that the wellbeing programs designed for schools may be inadequate for young athletes in talent development pathways. There are competition and performance implications of investing in mental wellbeing, as identified in the wider scientific literature and in international sporting associations such as that in the International Olympic Committee Consensus Statement on mental health in elite athletes (3). Early life is a known period for building competencies to support wellbeing that can track into adulthood. These findings may support the development of such competencies through mental wellbeing programs, and particularly initiatives that align with positive psychology. As per best practice guidelines for program development and evaluation, there remains the imperative that such programs are developed utilizing the correct expertise (which includes the lived experiences of the athletes themselves) to ensure validity, safety, efficacy, and rigor. This current work is particularly novel as it sits within a broader wellbeing program specifically designed for young athletes, and through which continuous improvement occurs as a result of these current findings. There is a need for athlete wellbeing programs to be both evidence informed, as well as developed based on the needs and experiences of those young people to which the program is targeted. That includes nuanced wellbeing programming for young women athletes, indeed based on the current data and supported by the wider literature. The socio-historical-cultural context of football in Australia provides further rationale for specific understanding of the young men and women's experiences given the women's league only recently become semi-professional relative to longer standing professional competition of the men's league (5).

## 5 Conclusion

The study is the first of its kind in terms of focusing on mental wellbeing informed by positive psychology, and the largest in terms of young Australian athletes in talent pathways. The findings suggest that mental wellbeing may present differently among athletes relative to the general population, and that may be particularly so for domains of connectedness and perseverance. These findings are somewhat unsurprising given the context of elite sport which necessitates specific skills and competencies. Further research is needed to confirm these differences which may help to inform future mental health promotion programs. Active appraisal, such as that achieved through this current study which is one part of a cyclical continuous improvement process, allows wellbeing programs to be bespoke to the needs for young people, through which improved outcomes may be enhanced.

## Data Availability

The datasets presented in this article are not readily available because they were collected under quality assurance and continuous improvement and are therefore not available by request to the corresponding author. Requests regarding the datasets should be directed to erin.hoare1@deakin.edu.au.
