# Supplementary material for: Promoting mental wellbeing among youth Australian Rules footballers through a model of continuous improvement
Source: Front Sports Act Living. 2024 Aug 9;6:1189933. doi: 10.3389/fspor.2024.1189933 (PMC11341484; doi:10.3389/fspor.2024.1189933)
Supplement: Supplementary file 1 [file Table1.docx]

***Supplementary Table 1*** *EPOCH data (mean, standard deviation) from AFL talent cohort and EPOCH student data from Kern et al., 2016*

|  | NAB Boys (n=299)  M(SD) | Student boys | Girls (n=309)  M(SD) | Student girls |
| --- | --- | --- | --- | --- |
| Engagement | 3.56 (0.75) | 3.30 (0.78) | 3.50 (0.79) | 3.18 (0.89) |
| Perseverance | 4.19 (0.58) | 3.63 (0.81) | 4.05 (0.67) | 3.50 (0.91) |
| Optimism | 3.66 (0.73) | 3.51 (0.79) | 3.51 (0.76) | 3.48 (0.92) |
| Connectedness | 4.40 (0.60) | 4.06 (0.78) | 4.39 (0.64) | 4.35 (0.82) |
| Happiness | 4.04 (0.70) | 3.85 (0.83) | 3.91 (0.81) | 3.96 (1.01) |
| Overall | 19.85 (2.47) | 18.35 (3.99) | 19.35 (2.77) | 18.47 (4.55) |
